# Supplementary material for: Axial de-scanning using remote focusing in the detection arm of light-sheet microscopy
Source: Nat Commun. 2024 Jun 12;15:5019. doi: 10.1038/s41467-024-49291-0 (PMC11169345; doi:10.1038/s41467-024-49291-0)
Supplement: Supplementary file 3 — Description of Additional Supplementary Files [file 41467_2024_49291_MOESM3_ESM.pdf]

Description of Additional Supplementary Files

File Name: **Supplementary Video 1**

Description: the light-sheet region translates along its width direction, corresponding to the Y-axis, while simultaneously being scanned in the axial or Z-axis, relative to the detection objective. As a result of this translation, the light-sheet region that must be cropped also shifts along the Y-axis. This dynamic adjustment ensures precise alignment and proper imaging throughout the scanning process.
